# Reactivation of *FMR1* by CRISPR/Cas9-Mediated Deletion of the Expanded CGG-Repeat of the Fragile X Chromosome

**DOI:** 10.1371/journal.pone.0165499

**Published:** 2016-10-21

**Authors:** Nina Xie, He Gong, Joshua A. Suhl, Pankaj Chopra, Tao Wang, Stephen T. Warren

**Affiliations:** 1 Department of Human Genetics, Emory University School of Medicine, Atlanta, Georgia, United States of America; 2 Departments of Biochemistry and Pediatrics, Emory University School of Medicine, Atlanta, Georgia, United States of America; Centre National de la Recherche Scientifique, FRANCE

## Abstract

Fragile X syndrome (FXS) is a common cause of intellectual disability that is most often due to a CGG-repeat expansion mutation in the *FMR1* gene that triggers epigenetic gene silencing. Epigenetic modifying drugs can only transiently and modestly induce *FMR1* reactivation in the presence of the elongated CGG repeat. As a proof-of-principle, we excised the expanded CGG-repeat in both somatic cell hybrids containing the human fragile X chromosome and human FXS iPS cells using the CRISPR/Cas9 genome editing. We observed transcriptional reactivation in approximately 67% of the CRISPR cut hybrid colonies and in 20% of isolated human FXS iPSC colonies. The reactivated cells produced FMRP and exhibited a decline in DNA methylation at the *FMR1* locus. These data demonstrate the excision of the expanded CGG-repeat from the fragile X chromosome can result in *FMR1* reactivation.

## Introduction

Fragile X syndrome (FXS) is the most common cause of inherited intellectual disability and is one of the leading monogenic causes of autism [1]. FXS is typically due to an expansion mutation of a CGG-repeat in the 5’-untranslated region (5’UTR) of the *FMR1* gene where, in contrast to the common normal repeat length of 30 triplets, FXS alleles expand well beyond 200 triplets [2]. *FMR1* alleles with this expanded repeat are referred to as the full mutation. In a response to the expanded repeat, the *FMR1* gene undergoes locus-specific hypermethylation and chromatin remodeling that epigenetically silences the gene [3–5]. Although it remains unclear how the expanded CGG-repeat results in the epigenetic silencing of the full mutation, several models have been proposed, including structural repeat changes in the DNA [6, 7], the mRNA [5, 8–12], or the formation of a DNA:RNA R-loops [13–15]. Regardless, the expanded full mutation CGG-repeat seems to be the prerequisite trigger to initiate and maintain repressive epigenetic changes in *FMR1*.

Since the CGG-repeat is in the 5’UTR and would not influence the coding region of *FMR1*, strategies to reactivate *FMR1* in FXS cells have been attempted. Small molecules that inhibit DNA methyltransferases [16, 17] or the histone deacetylases [4, 5] have been found to modestly increase *FMR1* transcription. However, such reactivation is typically only transient, and re-silencing of *FMR1* happens within days. Moreover, long-term use of such inhibitors imposes serious deleterious effects on the cells, which is potentially due to unnecessary expression of other genes caused by these small molecules.

A site specific genomic editing tool, the CRISPR (clustered regularly interspaced short palindromic repeats) system, has recently been developed and implemented to target and mutate specific genomic regions[18–21]. The CRISPR system is adapted from the native type II CRISPR system, which functions as an immune defense system in bacteria[18, 22, 23]. The CRISPR system has been applied in eukaryotic genome editing, in which the Cas9 protein and a single-guide RNA (sgRNA) are delivered into the cells of interest[24]. Once the sgRNA finds the target sequence, the Cas9 protein will generate a double strand break (DSB), and the cut site will be repaired through non-homologous end joining (NHEJ)[25].

In this work, we utilized CRISPR genome editing technology to excise the expanded CGG-repeat from the full mutation allele in FXS cells resulting in an *FMR1* allele without CGG-repeats. We hypothesized that excision of the expanded CGG-repeat from the *FMR1* full mutation may lead to constant *FMR1* reactivation. Initially, to ease cell culture and enhance cell cycling, we utilized a somatic hybrid cell line (Y75) that contains a single human fragile X chromosome in a rodent CHO cell line [26]. Successful excision of the full mutation resulted reactivation of *FMR1* transcription and translation in about half of the excised colonies. Similarly, we implemented the CRISPR experiments in human induced pluripotent stem (iPS) cells derived from a FXS patient [3]. After successful excision of the full mutation, we found one in five derived clonal lines had both transcriptional and translational reactivation, suggesting a successful approach for reactivation of *FMR1* in fragile X syndrome.

## Results

### CRISPR/Cas9 deletion of the CGG repeat tract

**Fig 1:**
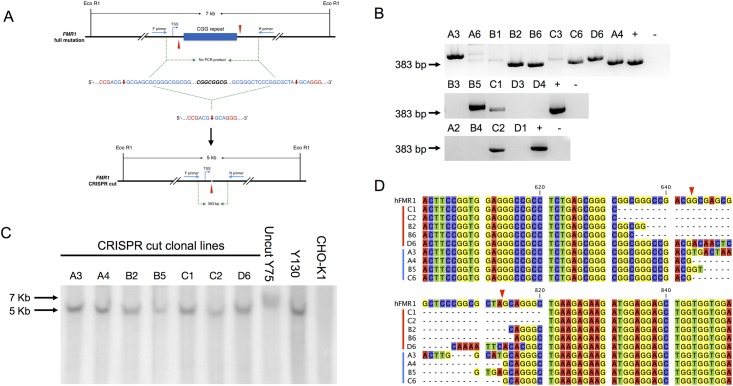
CRISPR/Cas9 mediated *hFMR1* CGG repeat removal in somatic hybrid CHO cell line. (A) The schematic view of the cleavage induced by CRISPR/Cas9 at the CGG repeat region. The double strand cut sites are marked by red arrows. The PAM sequence is shown in red. Primers used in PCR to verify the CGG repeat removal are indicated. EcoRI is the restriction enzyme site used for Southern blot. (B) PCR screening of colonies after FACS sorting. (C) Southern blot analysis of PCR positive colonies. (D) Sequence alignment of 9 PCR positive colonies. Theoretical breakpoints are indicated by red arrowheads.

We first designed and cloned a pair of single guide RNA (sgRNA) oligos targeting either side of the CGG repeat tract of *FMR1* into the backbone vector PX458 containing the GFP and Cas9 cassettes to construct two plasmids, named SW59 and SW60 (S1 Fig). Cells transfected with the two plasmids are expected to be transiently GFP positive, which can therefore be enriched by Fluorescence-activated cell sorting (FACS). Since the length and CG-content of the full mutation precludes conventional PCR across the repeat, the status of the CGG repeat can be simply assessed by the presence or absence of an amplicon, reflecting the CRISPR cut allele or the parental full mutation allele, respectively (Fig 1A). To validate the protocol in a human cell line, we co-transfected plasmids SW59 and SW60 into human HEK293FT cells and demonstrated efficient excision of normal CGG repeat allele while leaving the transcriptional start site intact (S2 Fig).

### Isolation of multiple CRISPR cut hybrid cell lines

Following transfection and FACS sorting of Y75 cells, a somatic hybrid cell line with a single human fragile X chromosome, serial dilution was used where cells are successively subdivided until a pure line is isolated [25]. Eighteen pure colonies from the sorted population of transfected cells were isolated. PCR across the human CGG-repeat identified 9 colonies with a PCR product in the expected size range, indicative of a presumed CRISPR cut *FMR1* allele (Fig 1B). To verify this, Southern blot analysis was performed by cleaving genomic DNA from the PCR positive colonies with EcoRI. As shown in Fig 1A, such cleavage of the full mutation would result in a 7 kb EcoRI fragment, where the normal allele would yield a 5 kb fragment. As expected, a 5 kb band was observed in the PCR positive colonies (Fig 1C) rather than a 7 kb band, indicating that CRISPR successfully removed the CGG-repeat.

To determine the precise breakpoint following CRISPR cleavage and NHEJ, sequence across the breakpoints was determined from the amplicon of each of the 9 somatic hybrid cell clonal lines. The actual breakpoints were within an average of 6 nucleotides from the theoretical breakpoint (Fig 1D). Thus, the CRISPR cleavage of the CGG-repeat was reasonably close to the predicted breakpoints with three of the hybrids showing some sequence changes on either side of the breakpoints or both (A3, B5, and D6). It is interesting to note that there are only 2 hybrids (A4 and C6) that have the resolved breakpoints at the predicted site.

### Expression of *FMR1* in CRISPR cut hybrid cell clonal lines

**Fig 2:**
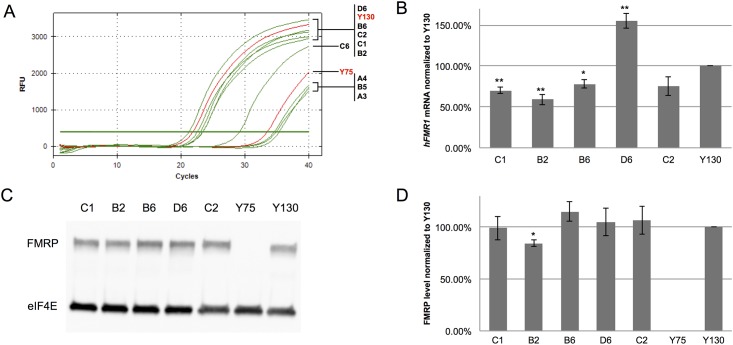
Reactivation of *hFMR1* in CRISPR cut clonal lines derived from somatic hybrid CHO cells. (A) Amplification view of the *hFMR1* signals of CRISPR cut clonal lines in qPCR. (B) qPCR data analysis of the human *FMR1* gene expression in CRISPR cut clonal lines. *Eif4e* gene expression was used as the internal control. *hFMR1* expression was further normalized to Y130. Error bars represent SE (n = 4 independent experiments). **p<0.01, *p<0.05. (C) Western blot analysis of FMRP in CRISPR cut clonal lines with reactivation of *hFMR1*. eIF4E was used as a loading control. (D) Densitometric analysis of FMRP levels in CRISPR cut clonal lines with reactivation of *hFMR1*. FMRP was normalized to eIF4E and further normalized to Y130. Error bars represent SE (n = 3 independent experiments). **p<0.01.

After confirming the successful cleavage by CRISPR in the somatic hybrid cells, we analyzed the expression of *FMR1* gene in the 9 confirmed clonal lines. 3 out of the 9 deletion positive clones remained the same expression level as Y75 (Fig 2A). 5 clones showed restored levels of human *FMR1* transcription when comparing to Y130, the hybrid cell line containing a normal human X chromosome (Fig 2A). Specifically, the average reactivation level of *FMR1* transcription was 87.6%. *FMR1* transcription levels of clones C1, B2 and B6 are statistically lower than that of Y130, whereas clone D6 has a significantly higher level than Y130 (Fig 2B). We also detected human FMRP expression in all 5 clones with reactivated *FMR1* transcription using the human specific FMRP antibody (Fig 2C). All reactivated clones except for B2 have similar FMRP expression levels when compared to Y130. Taken together, these data demonstrated that under certain circumstances excision of full mutation CGG repeat from the human fragile X chromosome could spontaneously lead to transcriptional and translational reactivation of *FMR1*.

### Isolation of a CRISPR cut FXS iPSC line with reactivation

**Fig 3:**
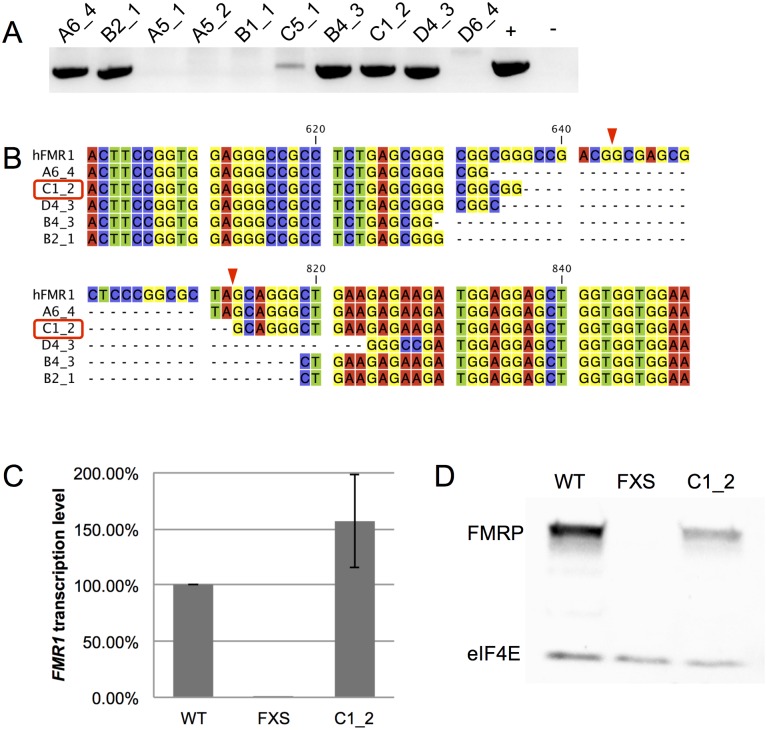
Identification of the CRISPR cut iPSC clonal line with *FMR1* reactivation. (A) PCR screening of iPSC colonies after FACS sorting. (B) Sequence alignment of 5 PCR positive colonies. (C) qPCR data analysis of *FMR1* gene expression in CRISPR cut clonal line C1_2 with reactivation. β-actin was used as the internal control. Error bars represent SE (n = 3 independent experiments). (D) Western blot analysis of FMRP in CRISPR cut clonal line C1_2 with reactivation of *FMR1*. eIF4E was used as a loading control.

Based on the results of reactivation of *FMR1* after CRISPR cleavage in somatic hybrid clonal lines, we further tested whether the CRISPR system could reactivate full mutation *FMR1* gene in a human FXS iPSC line. iPSCs nucleofected with the two CRISPR plasmids, SW59 and SW60, were sorted by FACS and seeded onto the irradiated MEF layer. We identified 5 CRISPR cut clonal lines and confirmed the removal of CGG repeats by CRISPR deletion genotyping PCR (Fig 3A). Identification of the exact cleavage site of these 5 positive clones revealed that, unlike the somatic hybrid cells, the first breakpoint was about 10 nucleotides upstream of the theoretical cut site, whereas the second breakpoint varied among all 5 clones (Fig 3B). Clone C1_2 showed both transcriptional and translational reactivation and sustained stable reactivation level after being cultured for 50 days (Fig 3C and 3D). Again, the data suggested that the excision of full mutation CGG repeats from human iPS cells was able to reactivate *FMR1* gene.

### DNA Methylation profiling of CRISPR cut hybrid cell lines and FXS iPSC lines

**Fig 4:**
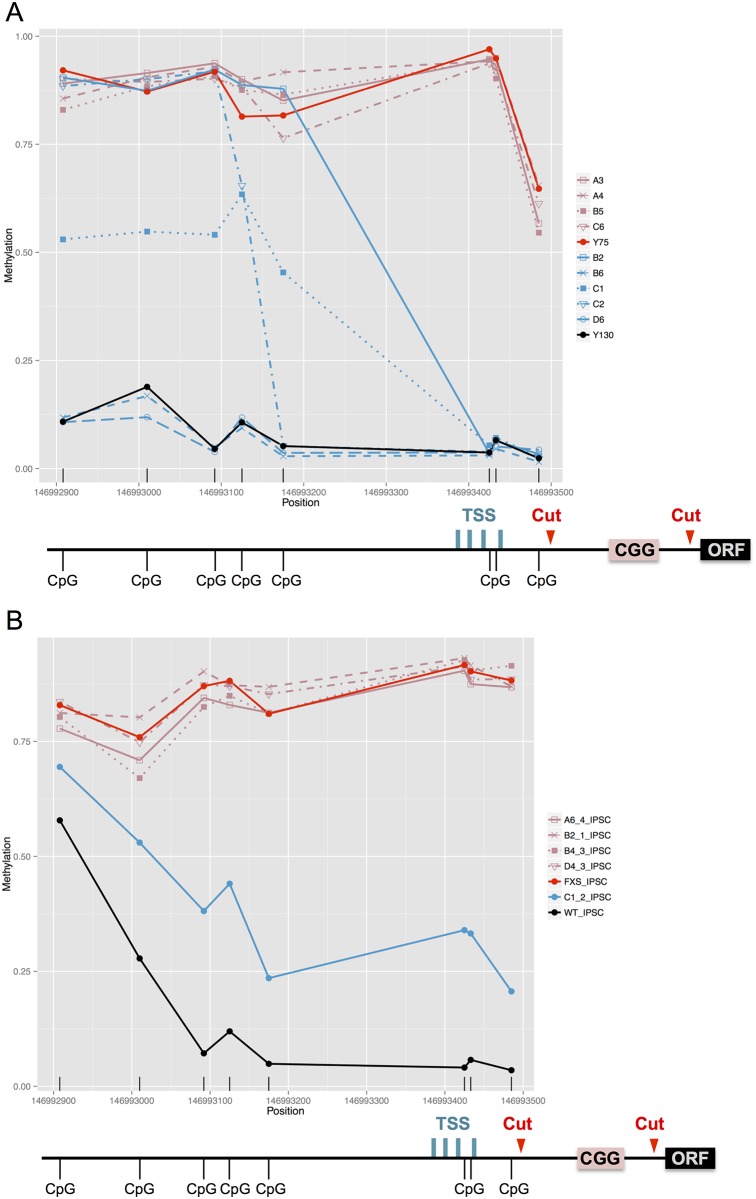
Methylation profiling analysis of CRISPR cut clonal lines derived from somatic hybrid CHO cells and iPS cells. DNA methylation profiling analysis was performed using Infinium HumanMethylation450 BeadChip. Data analysis was focused on the 8 probe positions at the promoter region of *FMR1* gene. Silent clonal lines were shown in red, and expressing clonal lines were shown in blue. (A) Methylation states of CRISPR cut somatic hybrid CHO cells. (B) Methylation states of CRISPR cut iPS cells.

After observing that not all CRISPR cut clonal lines are capable of reactivating *FMR1*, we considered that the expression of *FMR1* correlated with the epigenetic status of the promoter region after the CGG repeat removal. We analyzed the methylation state of *FMR1* promoter in both reactivated and non-reactivated clonal lines of the somatic hybrid cell line and FXS iPS cells using the Infinium HumanMethylation450 BeadChip Kit. Methylation profiles for the probes that are in the promoter region were generated based on the methylation values at each CpG site for the CRISPR cut clonal lines of the somatic hybrid cell line A3, A4, B2, B5, B6, C1, C2, C6, and D6 (Fig 4A). CRISPR cut non-reactivated clonal lines closely represent the hypermethylation status as the FXS control line Y75. Among the clonal lines with reactivation, two had similar methylation level as the WT control line Y130 at all 8 probe positions in the promoter region. The rest of the reactivated clonal lines had relatively high methylation levels detected in the upstream several CpG sites, and the methylation levels decreased dramatically at the CpG sites adjacent to the CGG repeat region. Analysis of the methylation profiles of the CRISPR cut FXS iPSC clonal lines at the *FMR1* promoter regions revealed that the non-reactivated clonal lines remained highly methylated as the uncut FXS iPS cells (Fig 4B). The clonal line with reactivation exhibited a significant decrease of methylation across the promoter region. Although not as low as the WT iPSC control line, the decreased methylation level is sufficient for *FMR1* expression. All together, the methylation profile of each CRISPR cut clonal line is consistent with its respective *FMR1* expression status.

## Discussion

We demonstrated the capacity of CRISPR/Cas9 system to excise the full mutation CGG repeat of the *FMR1* gene in multiple cell lines, including human FXS iPS cells. Importantly, we showed that CRISPR/Cas9 mediated full mutation CGG repeat deletion could spontaneously reactivate *FMR1* thereby restoring FMRP production.

Not all CRISPR-cut colonies exhibited *FMR1* reactivation following the full mutation CGG repeat deletion. The most likely explanation for the reactivation observed in our experiments may be due to cell replication following CRISPR cleavage. Immediately following DNA replication the DNA is hemimethylated and the methylation pattern is restored by the maintenance methyltransferase DNMT1 which methylates the CpG sites of the newly synthesized strand opposite the pre-existing methylated cytosine (5mC) [27]. After the CGG repeat removal in those CRISPR cut clones, the signal that directs DNMT1 to methylate the unmethylated DNA strand may be weakened or slowed such that mitotic replication is completed without complete remethylation of the newly synthesized strand. This may also explain why a higher reactivation rate is observed in the somatic hybrid cells. That is, the somatic hybrid cells divide faster than the iPSCs, so there may be a higher chance for the somatic hybrid cells to lose methylation. It will be important to determine if similar CRISPR deletion of the full mutation repeat in nondividing neurons reactivates *FMR1*.

While this work was being concluded, Park et al. (Cell Rep Oct 13 2015) reported similar work using CRISPR excision of the full mutation with reactivation of *FMR1*. However, they induced a single double strand break (DSB) near the CGG repeat and relied upon somewhat random nonhomologous end joining to excise the repeat. Thus, the precision of the CRISPR deletion was not as precise as our approach of creating two flanking DSB. Similar to our observations, Park et al. also observed reactivation of *FMR1*, although differences in experimental details make frequency comparisons between the two studies difficult. However, the results presented above validate that CRISPR deletion of the full mutation CGG repeat on the fragile X chromosome can reactivate the silenced *FMR1* gene.

## Materials and Methods

### Cell line maintenance

HEK293FT was maintained in DMEM supplemented with 10% FBS and 1% P/S at 37°C with 5% CO2 incubation. Somatic hybrid cell lines Y75 and Y130 were maintained in HAS medium (DMEM supplemented with 10% FBS, 1% P/S, 1 μM L-Proline, 10 μM azaserine, and 100 μM hypoxanthine) at 37°C with 5% CO2 incubation. iPSCs were maintained in the feeder-free culture system following the protocol from STEMCELL Technologies. iPSCs were fed daily with mTeSR1 (STEMCELL Technologies, Vancouver, Canada) on matrigel (Corning, Tewksbury, MA) coated plate and were passed every 5–7 days using the enzymatic free method with ReLeSR (STEMCELL Technologies Vancouver, Canada).

### Transfection

HEK293FT cells were transfected using Lipofectamine 3000 (Thermo Fisher Scientific, Waltham, MA). Specifically, 0.5 x 10^6^ cells were seeded into one well of a 6-well plate one day prior to transfection. 2 μg CRISPR plasmids and 2 μl P3000 was diluted in 125 μl opti-MEM (Thermo Fisher Scientific, Waltham, MA) and was further mixed with 4 μl lipofectamine 3000 diluted in 125 μl opti-MEM for each well to be transfected. After 5 min incubation at room temperature, the transfection reagent mixture was added evenly into the well. 48–72 h post transfection, cells were ready to be harvested for other downstream experiments.

Somatic hybrid cell line Y75 was transfected using Lipofectamine LTX (Thermo Fisher Scientific, Waltham, MA). Specifically, 0.2 x 10^6^ cells were seeded into one well of a 6-well plate one day prior to transfection. 1h prior to transfection, complete HAS medium was switched to antibiotic-free HAS medium (2 ml/well). 2 μg CRISPR plasmids diluted in 250 μl opti-MEM was mixed with 4 μl LTX diluted in 250 μl opti-MEM for each well to be transfected. After 25 min incubation at room temperature, the transfection reagent mixture was added evenly into the well. 24 h post transfection, cells were ready for FACS. 48–72 h post transfection, cells were ready to be harvested for other downstream experiments.

iPSCs were transfected using nucleofection method. Specifically, 1 d prior to the passage day, iPSCs were treated with 10 μM Y-27632 overnight. On passage day, 4 x 10^6^ iPSCs resuspended in human stem cell nucleofector solution 1(Lonza, Basel, Switzerland) were nucleofected with 6 μg CRISPR plasmids (SW59+SW60) using program B16 in the nucleofector IIb device (Lonza, Basel, Switzerland). After being transferred to a freshly coated matrigel plate, the nucleofected cells were fed with mTeSR1 containing 10 μM Y-27632 for 1 d followed by regular mTeSR1 medium.

### DNA extraction

For genotyping, DNA was extracted using Epicentre QuickExtract^™^ DNA extraction solution (Epicentre, Madison, WI) following the manufacturer’s protocol. We use 100 μl or 400 μl of the solution for each well of a 24-well or 6-well plate, respectively. For Southern blot, DNA was extracted using QIAGEN DNeasy Blood & Tissue Kit (QIAGEN, Hilden, Germany) following the manufacturer’s protocol.

### CRISPR deletion genotyping

The CRISPR cut allele was amplified using TaKaRa LA *Taq* DNA polymerase (Clontech Laboratories, Mountain View, CA). 2 μl DNA template in Epicentre QuickExtract^™^ DNA extraction solution was mixed with 12.5 μl 2x GC buffer I, 1 μl 10 μM primer CRISPR Del_F (5’-GGAGGGAACAGCTTGATCAC-3’), 1μl 10 μM primer CRISPR Del_R (5’-ACTGGACTTGGGGCCTGTT-3’), 4 μl dNTP mixture, 0.25 μl LA Taq and 4.25 μl nuclease-free water to a final volume of 25 μl, and further subjected to a thermocycler program as follows: 94°C for 5 min, (94°C for 30 s, 60°C for 30 s, 72°C for 30 s) x 35 cycles, 72°C for 5 min, 4°C hold. PCR products were analyzed on a 1.5% agarose gel.

### Isolation of clonal lines

Transfected Y75 cells sorted on a Becton Dickinson FACS Aria II cell sorter were first seeded into a 24-well plate at the density of 5 cells/well. 7 days after the seeding single or mixed colonies would appear. 14 days after the seeding cells were ready to make replica plates for genotyping and human specific *FMR1* qPCR. Genotyping positive colonies were further seeded into a 96-well plates at the density of 0.5 cell/well to isolate morphologically single colonies. After confirming purified single colonies by genotyping, colonies were viewed as pure CRISPR cut clonal lines.

Transfected iPS cells sorted on a Becton Dickinson FACS Aria II cell sorter were first seeded onto a MEF layered 24-well plate at the density of 100 cells/well. The MEF coating density was 4 x 10^4^ cells/cm^2^. iPS cell aggregates appear between d7 and d10 post sorting. At this time point, pour a new layer of MEF to coat the plate again to substitute the old MEF layer. Continue feeding the cells until iPSC colonies were big enough for toothpick PCR based CRISPR deletion genotyping. Deletion positive colonies were transferred to the matrigel plate and cultured as pure CRISPR cut clonal lines.

### Southern blot

A total of 8 μg genomic DNA was fast digested with 5 μl EcoRI, 5 μl PteI, 5 μl 10x buffer and nuclease free water in a 50 μl reaction system at 37°C for 2 h. The digested DNA fragments were separated on a 0.8% agarose gel and transferred to nylon membrane. Prehybridize the membrane with 600 μl salmon sperm and 14 ml hybridization buffer (add 350 ml 20% SDS stock, 75 ml 20x SSC, 100 g PEG8000, and 250 mg heparin into 400 ml 80°C water, then add more water to a final volume of 1 L) at 65°C for 3–4 h. During prehybridization, prepare p32 labeled probe (primers for amplifying probes are listed in S1 Table) following Invitrogen RadPrime DNA labeling system (Thermo Fisher Scientific, Waltham, MA). The reaction mixture was purified through Sephadex column by centrifugation at 1700 rpm for 2 min. Replace prehybridization buffer with 10 ml hybridization buffer containing 400 μl salmon sperm and purified p32 labeled probe for overnight hybridization. The next day, the membrane was washed twice briefly with 25 ml Buffer I (mix 5 ml 20% SDS and 100 ml 20x SSC with 895 ml water) at room temperature, followed by a 15 min wash with 50 ml 65°C Buffer I at 65°C, and another 30 min wash with 50 ml 65°C Buffer II (mix 25 ml 20% SDS and 5 ml 20x SSC with 970 ml water) at 65°C. Expose the membrane to storage phosphor screen overnight before developing the film for an image.

### RNA extraction and RT-PCR

RNA was extracted using Trizol LS (Thermo Fisher Scientific, Waltham, MA) following manufacturer’s protocol. iTaq universal SYBR Green one-step kit (Bio-Rad, Hercules, CA) was used for human specific qPCR assay. 4 μl RNA template (50 ng/μl) was mixed with 10 μl 2x iTaq universal SYBR Green reaction mix, 0.6 μl 10 μM primer hs_FMR1 qPCR F (5’-AGAGGACAAGGAGGAAGAGGACGT-3’), 0.6 μl 10 μM primer hs_FMR1 qPCR R (5’-CTTTACCCGTGCGCAGCCGAC-3’), 0.25 μl iScript reverse transcriptase, 4.55 μl nuclease-free water to a final volume of 20 μl and further subjected to a thermocycler program in Bio-Rad CFX96 as follows: 50°C for 10 min, 95°C for 1 min, (95°C for 10 s, 55°C for 30 s) x 40 cycles, 95°C for 1 min, 55°C for 1 min, melt-curve analysis from 55°C to 95°C with 0.5°C increment/10 s. Control primers are listed in S1 Table. Samples from duplicate or triplicate wells in each experiment were viewed as technical repeats and were averaged as one independent data point. Unpaired student’s t-test was used for comparison between two different data sets. Only samples harvested from different experiments were viewed as biological repeats.

### Western blot

Cells were lysed in 50–100 μl lysis buffer per well of a 6-well plate for 30 min on ice. Lysate was clarified by centrifugation at 14000 rpm for 10 min. A total of 30–40 μg protein for each sample was separated on 4–15% Mini-PROTEAN TGX Stain-Free^™^ Precast Gels (Bio-Rad, Hercules, CA) at 200 V for 30 min, and transferred to PVDF membrane using Trans-blot Turbo Transfer System (Bio-Rad, Hercules, CA) at 1.3 A, 25 V for 10 min. Membrane was blocked with StartingBlock T20 (PBS) blocking buffer (Thermo Fisher Scientific, Waltham, MA) at room temperature for 15 min followed by incubating with human specific anti-FMRP primary antibody (6B8) (BioLegend, San Diego, CA) or anti-eIF4E primary antibody (BD Biosciences, San Jose, CA) at 4°C overnight. The next day, the membrane was washed with blotto buffer (mix 50 ml 10x PBS, 5 g non-fat milk powder, and 1 ml Tween 20 in 450 ml water) for 5 min at room temperature followed by incubation with anti-mouse secondary antibody for 1 h at room temperature. After 3 times of 10 min washes with blotto buffer at room temperature, the membrane was incubated with the clarity western ECL substrate solution (Bio-Rad, Hercules, CA) for 5 min and exposed with the ChemiDoc Touch imaging system (Bio-Rad, Hercules, CA). Image analysis was performed using Image Lab^™^ Software (Bio-Rad, Hercules, CA).

### Sequencing alignment

Sequencing alignment was done using CLC sequence viewer 7.0.2. Human *FMR1* sequence used for experimental design and alignment was from Human Assembly Feb. 2009 (GRCh37/hg19) at position: chrX:146992469–147032647.

## Supporting Information

S1 Fig CRISPR plasmid design schematic. (PDF)

S2 Fig Validation of CRISPR plasmid constructs SW59 and SW60 in 293FT cells. (PDF)

S1 Table List of primer sequences. (PDF)
