# Supplementary material for: Reactivation of FMR1 by CRISPR/Cas9-Mediated Deletion of the Expanded CGG-Repeat of the Fragile X Chromosome
Source: PLoS One. 2016 Oct 21;11(10):e0165499. doi: 10.1371/journal.pone.0165499 (PMC5074572; doi:10.1371/journal.pone.0165499)
Supplement: S1 Fig — (PDF) [file pone.0165499.s001.pdf]

Supplementary Figure 1

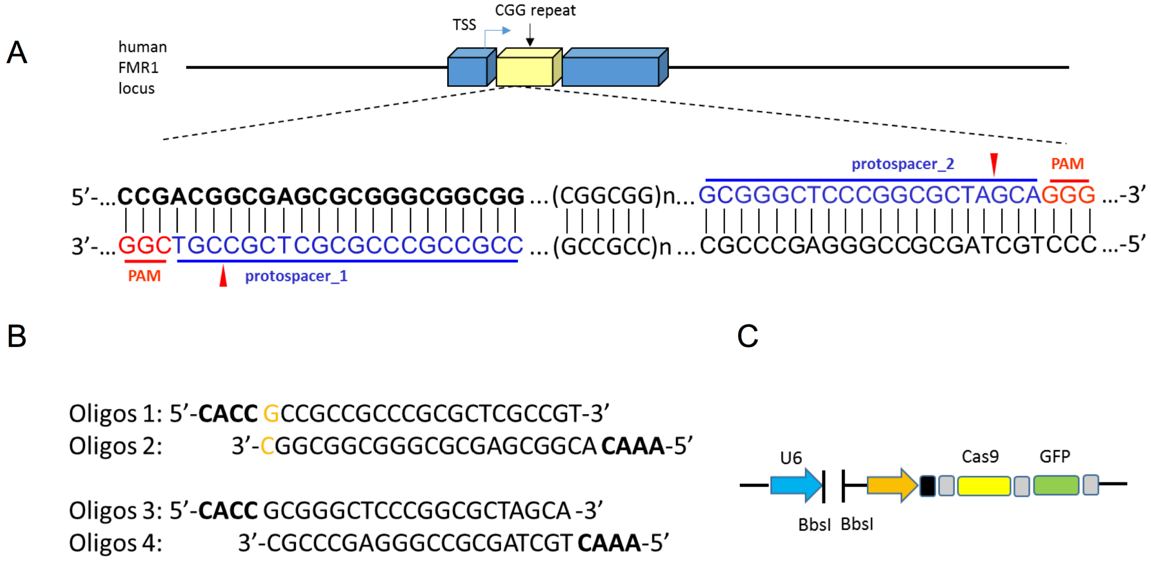

S1 Fig. CRISPR plasmid design schematic.

(A) sgRNA sequences were designed using the on-line tool: http://crispr.mit.edu/. The target sequences in the genome are shown in blue. PAM sequences are shown in red. The two red arrows indicate the CRISPR cut sites.

(B) The two pairs of sgRNA oligos were inserted into the BbsI site of the vector backbone expressing Cas9 and GFP (Addgene No. 48138) to make the two CRISPR plasmids named SW59 and SW60. The plasmid driven by U6 promoter requires a G at the TSS. An additional G shown in orange color is added into the first sgRNA sequence.

(C) Schematic of the sgRNA vector backbone PX458.
