# Supplementary material for: Reactivation of FMR1 by CRISPR/Cas9-Mediated Deletion of the Expanded CGG-Repeat of the Fragile X Chromosome
Source: PLoS One. 2016 Oct 21;11(10):e0165499. doi: 10.1371/journal.pone.0165499 (PMC5074572; doi:10.1371/journal.pone.0165499)
Supplement: S2 Fig — (PDF) [file pone.0165499.s002.pdf]

Supplementary Figure 2

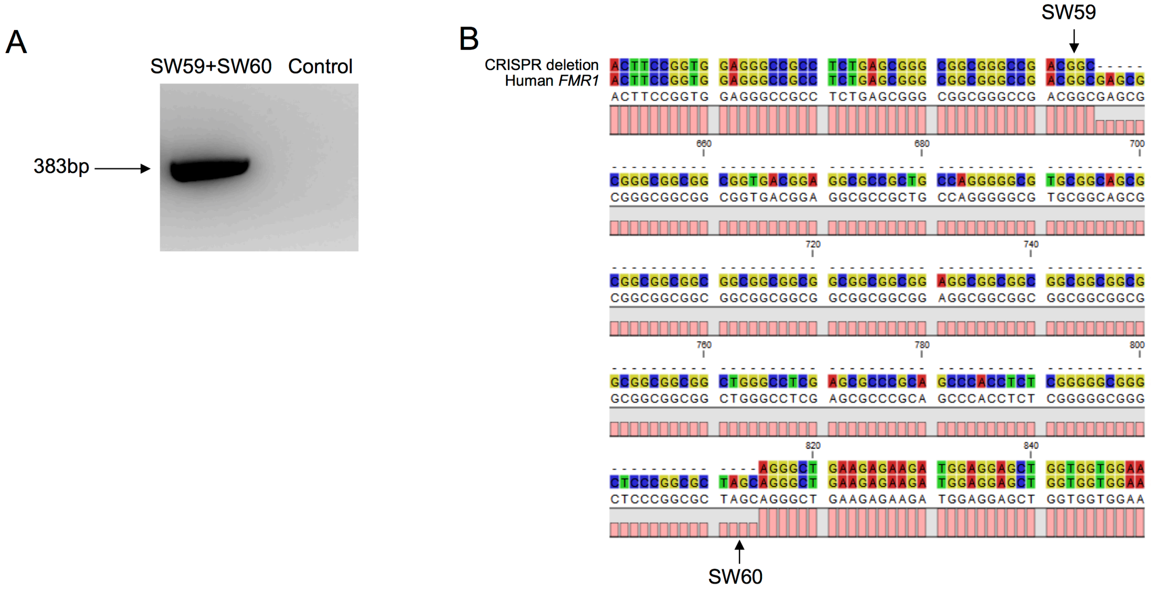

S2 Fig. Validation of CRISPR plasmid constructs SW59 and SW60 in 293FT cells. (A) CRISPR deletion genotyping for 293FT co-transfected with SW59 and SW60. (B) Sequencing alignment between CRISPR deletion amplicon and human *FMR1* gene showed that co-transfection of the two plasmids can induce efficient CGG repeats excision 293FT while keeping the TSS intact. Theoretical breakpoints were indicated by the arrows.

S1 Table. List of primer sequences

Primer	Sequence(5’-3’)
CRISPR del F	GGAGGGAACAGCGTTGATCAC
CRISPR del R	ACTGGACTTGGGGCCTGTT
PX458 sequencing primer	GGACTATCATATGCTTACCGTAACTTGA
Southern probe F	CTTCTCAGTTGGATACCAGCA
Southern probe R	CCACCGGAAGTGAAACCG
Hs_FMR1 qPCR F	AGAGGACAAGGAGGAAGAGGACGT
Hs_FMR1 qPCR R	CTTTACCCGTGCGCAGCCGAC
eIF4E qPCR F	GTGTCTGCATGGGACTGATAA
eIF4E qPCR R	AGACGCAGTCACACACATAG
Actin qPCR F	CTCTCCAGCCTTCCTTCCT
Actin qPCR R	AGCACTGTGTTGGCGTACAG
