# Supplementary material for: Reactivation of FMR1 by CRISPR/Cas9-Mediated Deletion of the Expanded CGG-Repeat of the Fragile X Chromosome
Source: PLoS One. 2016 Oct 21;11(10):e0165499. doi: 10.1371/journal.pone.0165499 (PMC5074572; doi:10.1371/journal.pone.0165499)
Supplement: S1 Table — (PDF) [file pone.0165499.s003.pdf]

S1 Table. List of primer sequences

Primer	Sequence(5'-3')
CRISPR del F	GGAGGGAACAGCGTTGATCAC
CRISPR del R	ACTGGACTTGGGGCCTGTT
PX458 sequencing primer	GGACTATCATATGCTTACCGTAACTTGA
Southern probe F	CTTCTCAGTTGGATACCAGCA
Southern probe R	CCACCGGAAGTGAAACCG
Hs_FMR1 qPCR F	AGAGGACAAGGAGGAAGAGGACGT
Hs_FMR1 qPCR R	CTTTACCCGTGCGCAGCCGAC
eIF4E qPCR F	GTGTCTGCATGGGACTGATAA
eIF4E qPCR R	AGACGCAGTCACACACATAG
Actin qPCR F	CTCTCCAGCCTTCCTTCCT
Actin qPCR R	AGCACTGTGTTGGCGTACAG
